# Epidemiological Profile of Exogenous Intoxications by Self‐Medication in Brazil: A Decade of Trends and the Impact of the COVID‐19

**DOI:** 10.1002/pds.70269

**Published:** 2025-11-16

**Authors:** Lucas Borges Pereira, Júlia Casanova Durante, Leonardo Régis Leira Pereira, Fabiana Rossi Varallo, Maria Olívia Barboza Zanetti

**Affiliations:** ^1^ Department of Pharmaceutical Sciences | School of Pharmaceutical Sciences of Ribeirão Preto University of São Paulo São Paulo Brazil; ^2^ Barretos School of Health Sciences Dr. Paulo Prata—FACISB São Paulo Brazil

**Keywords:** drug misuse, drug overdose, drug‐related side effects and adverse reactions, health information systems

## Abstract

**Purpose:**

Self‐medication carries the potential for significant adverse events when practiced irresponsibly. The indiscriminate use of medicines notably intensified during the COVID‐19 pandemic. This study aimed to investigate the epidemiological profile of exogenous intoxications due to self‐medication among Brazilians from 2014 to 2023.

**Methods:**

This was a cross‐sectional, descriptive, and exploratory study utilizing secondary data from the Brazilian Ministry of Health's Notifiable Diseases Information System. Confirmed cases of self‐medication intoxication reported between 2014 and 2023 were included. Descriptive analysis, incidence and lethality rate calculations, chi‐squared tests (*p* ≤ 0.05), and Multiple Correspondence Analysis (MCA) were performed to explore potential associations between sociodemographic and clinical variables.

**Results:**

A total of 23 859 cases were analyzed. The study observed a predominance of adults (20–59 years), women (70.8%), and individuals self‐identifying as White or Brown (mixed‐race). Most cases resulted from an acute‐single exposure to the medication and resolved with complete recovery without sequelae. There was a national increase in incidence, particularly in 2022 and 2023, and significant variations among Brazilian Federative Units. The MCA identified associations between advanced age and the type of exposure (repeated or chronic) and the severity of outcomes. It also revealed changes in the sociodemographic profile of self‐medication intoxications during the COVID‐19 pandemic.

**Conclusions:**

These findings underscore the pandemic's impact on self‐medication patterns and intoxication notifications. The study highlights the need for public policies focused on health education, appropriate medicine use, and strengthening the culture of reporting in Brazil.

**Summary**

- Reported self‐medication intoxications increased nationwide, with a sharp rise after 2022.
- Women, adults, and individuals self‐identifying as white or pardo were most affected.
- The COVID‐19 pandemic altered patterns, with more repeated and chronic exposures.
- Regional disparities reveal weaknesses in surveillance and notification systems.
- Older adults faced greater risk of severe outcomes, including fatal cases.

## 1 Introduction

Medicines are indispensable tools in modern healthcare, offering effective treatment, symptom relief, and disease prevention. However, even when appropriately used, medicines carry inherent risks, which are magnified by self‐medication practices [1]. According to the World Health Organization (WHO), self‐medication is defined as the selection and use of medicines by individuals to treat self‐diagnosed illnesses or symptoms, or the continued use of previously prescribed medicines without medical guidance [2].

Self‐medication is a global public health concern, with prevalence rates ranging widely from 11.7% to 92.0% across different populations. Several factors contribute to this behavior, beyond the pursuit of self‐care, including limited access to healthcare services, misconceptions about drug safety, the perception of pharmacies primarily as commercial establishments, aggressive pharmaceutical marketing, and the availability of medicines in nonpharmacy settings [3]. While responsible self‐medication can offer benefits, such as symptom relief and reduced healthcare costs, irresponsible practices lead to substantial risks, including drug abuse, adverse events, delayed diagnosis, antimicrobial resistance, and increased morbidity and mortality [3, 4, 5].

The COVID‐19 pandemic further intensified the risks of self‐medication. The widespread promotion and use of medicines with unproven efficacy, often endorsed by governmental and media entities, resulted in unprecedented increases in pharmacy sales and a surge in adverse drug events, including cases of intoxication [4, 6]. Although the global implications of pandemic‐driven self‐medication have been widely debated, evidence from low‐ and middle‐income countries remains limited, particularly regarding patterns of intoxication associated with this practice.

In this context, it is important for establishing public policies and actions to understand the profile of intoxications resulting from self‐medication in the Brazilian population, as well as the influence of the pandemic on this practice. This study aims to analyze the epidemiological profile of exogenous intoxications due to self‐medication in Brazil between 2014 and 2023.

## 2 Methods

This was a cross‐sectional, descriptive, and exploratory study based on reporting of exogenous intoxications due to self‐medication in Brazil over a decade. Data were obtained from the Notifiable Diseases Information System (SINAN), managed by the Brazilian Ministry of Health and publicly available through the DataSUS platform [7].

We included all confirmed cases of exogenous intoxication in which self‐medication was recorded as the circumstance of exposure between January 2014 and December 2023. Records that did not list medicines as the causative agents were excluded. Data collection was conducted between July and December 2024.

The variables analyzed included sociodemographic characteristics (age, education level, race, sex, pregnancy trimester), classification of exposure to intoxication (acute‐single, acute‐repeated, chronic, or acute on chronic), confirmation criterion (clinical‐laboratory, clinical‐epidemiological, clinical), outcome (cure without sequelae, cure with sequelae, death by intoxication, death by other causes, loss to follow‐up), as well as the year and federal unit (FU) of notification. For the purposes of analysis, the years 2020–2023 were considered the pandemic period, in order to evaluate the potential impact of COVID‐19 on self‐medication‐related intoxications.

To calculate incidence rates, the number of confirmed self‐medication intoxications was divided by the annual population estimate of the corresponding FU, multiplied by 100 000 inhabitants. Lethality rates were calculated as the number of deaths due to self‐medication intoxication divided by the total number of confirmed cases in the same FU and year, multiplied by 100. Population data were derived from official estimates of the Brazilian Institute of Geography and Statistics (IBGE).

Data were managed using Microsoft Office Excel and analyzed with Spyder software (version 5.5.1). Initially, exploratory data analysis was conducted, considering measures of central tendency and dispersion. Qualitative variables were summarized using absolute and relative frequencies. The association between categorical variables was explored using the chi‐squared test, adopting a significance level of 5%. The variables “education level” and “pregnancy trimester” were excluded from these analyses due to the unavailability of individual‐level data in DataSUS.

In the second stage, Multiple Correspondence Analysis (MCA) was applied to explore relationships between sociodemographic characteristics and intoxication features (type of exposure and outcome). MCA is a noninferential exploratory statistical technique that graphically represents categorical data in a multidimensional space, where the proximity of points indicates association. The contribution of each category to the formation of the axes is evaluated by absolute and relative contributions. This technique is particularly appropriate for studying population data and risk factors [8].

For MCA, categories were grouped as follows: year (prepandemic: 2014–2019; pandemic: 2020–2023); age (0–19, 20–59, ≥ 60 years); ethnicity (white, black, brown, others); sex (male, female); type of exposure (acute‐single, acute‐repeated, chronic, or acute on chronic); and outcome (cure without sequelae, cure with sequelae, death by intoxication, death by other causes, loss to follow‐up). Cases with unspecified information for any of these variables were excluded. Only variables with statistical significance (*p* ≤ 0.05) in the chi‐squared test were selected for MCA. The variable “Confirmation criterion” was excluded due to its limited epidemiological relevance and its negative effect on graphical representation.

## 3 Results

A total of 25 965 cases of exogenous intoxication due to self‐medication were reported between January 2014 and December 2023. After excluding 2106 records that did not list medicines as causative agents, 23 859 cases were included in the analysis, distributed as follows: 1656 in 2014, 1509 in 2015, 1580 in 2016, 1964 in 2017, 2503 in 2018, 2972 in 2019, 2332 in 2020, 2363 in 2021, 3174 in 2022, and 3806 in 2023.

Table 1 summarizes the sociodemographic profile of affected individuals. Most cases occurred among adults (62.2%, *n* = 14 842), females (70.8%, *n* = 16 896), individuals of white (44.3%, *n* = 10 570) or brown ethnicity (37.7%, *n* = 8987), and those with complete or incomplete high school education (27.5%, *n* = 6559). A total of 265 cases (1.1%) involved pregnant women. Missing data were particularly frequent for education level (44%, *n* = 10 492).

**TABLE 1:** Sociodemographic variables of individuals affected by self‐medication intoxication between 2014 and 2023 in Brazil.

Variables	Frequency (%)										
	2014	2015	2016	2017	2018	2019	2020	2021	2022	2023	2014–2023
Age (years)											
< 1	2.7	1.5	1.4	2.6	1.5	1.6	1.2	1.1	1.5	1.2	1.6
1–9	7.5	7.4	6.7	7.5	5.4	4.5	5.0	4.1	3.9	3.7	5.2
10–19	22.2	23.1	22.2	25.5	25.9	28.2	25.2	27.5	29.5	26.7	26.2
20–59	63.0	63.8	63.6	59.6	62.0	61.7	63.5	62.4	60.2	63.3	62.2
60–79	3.6	3.6	5.4	4.2	4.3	3.3	4.5	4.3	4.1	4.4	4.2
≥ 80	0.8	0.6	0.6	0.7	0.9	0.6	0.5	0.6	0.8	0.7	0.7
Not filled	0.0	0.0	0.0	0.0	0.0	0.0	0.0	0.0	0.0	0.0	0.0
Education level											
Illiteracy	1.0	1.2	1.0	0.7	0.7	0.6	0.5	0.3	0.5	0.4	0.6
1st–4th grade incomplete	5.0	4.2	5.1	3.8	4.0	3.6	3.8	2.8	2.8	3.3	3.7
4th grade complete	3.7	2.7	2.5	2.5	2.3	2.7	2.2	1.5	1.7	2.1	2.3
5th–8th grade incomplete	13.4	11.2	10.7	10.0	11.1	9.4	8.8	9.4	8.9	9.6	10.0
Elementary school complete	4.7	5.3	4.7	4.6	5.8	5.2	4.5	6.2	4.9	5.4	5.2
High school incomplete	7.9	9.6	7.2	11.6	9.9	10.6	10.2	10.2	10.4	10.8	10.1
High school complete	10.9	14.0	14.0	14.5	15.6	16.1	19.1	19.0	19.1	23.4	17.4
Higher education incomplete	1.6	2.3	2.6	2.9	3.0	3.6	3.1	3.5	3.3	3.6	3.1
Higher education complete	2.7	2.7	2.9	3.1	3.7	3.3	3.1	4.8	4.0	4.6	3.6
Not filled	48.9	46.7	49.3	46.3	43.9	44.9	44.7	42.4	44.4	36.8	44.0
Race											
White	40.7	44.8	42.4	45.8	45.1	43.2	43.6	44.6	43.8	46.6	44.3
Black	4.2	3.9	4.1	3.6	4.6	4.1	5.1	5.5	4.5	5.0	4.6
Asian	0.4	0.3	0.5	0.9	0.6	0.5	0.6	0.6	0.9	0.7	0.6
Brown	37.3	34.6	35.3	35.1	36.8	35.4	37.5	38.2	40.0	41.5	37.7
Indigenous	0.4	0.2	0.3	0.3	0.4	0.2	0.3	0.3	0.2	0.3	0.3
Not filled	17.1	16.2	17.5	14.5	12.4	16.6	12.9	10.8	10.7	5.8	12.6
Sex											
Male	32.1	32.7	31.5	31.6	28.8	27.9	28.6	27.5	26.9	28.7	29.2
Female	67.9	67.3	68.4	68.4	71.2	72.1	71.4	72.5	72.9	71.3	70.8
Not filled	0.0	0.0	0.1	0.0	0.0	0.0	0.0	0.0	0.1	0.0	0.0
Gestation											
1st trimester	0.3	0.3	0.3	0.3	0.6	0.3	0.2	0.3	0.4	0.3	0.3
2nd trimester	0.5	0.5	0.3	0.5	0.4	0.5	0.4	0.4	0.4	0.3	0.4
3rd trimester	0.2	0.2	0.1	0.2	0.2	0.3	0.3	0.3	0.3	0.2	0.2
Unknown	0.1	0.1	0.1	0.2	0.3	0.0	0.2	0.1	0.1	0.1	0.1
Nonpregnant	88.2	87.3	88.7	87.3	85.5	84.2	85.1	86.5	87.0	90.2	87.0
Not filled	10.7	11.7	10.6	11.6	13.0	14.6	13.8	12.4	11.7	8.9	11.9

As shown in Table 2, acute single exposure predominated (72.8%, *n* = 17 370). Most cases were confirmed using clinical criteria alone (70.8%, *n* = 16 885) and evolved to recovery without sequelae (84.9%, *n* = 20 255).

**TABLE 2:** Variables related to self‐medication intoxication between 2014 and 2023 in Brazil.

Variables	Frequency (%)										
	2014	2015	2016	2017	2018	2019	2020	2021	2022	2023	2014–2023
Type of exposure to intoxication											
Acute‐single	80.4	77.3	75.7	75.0	71.6	74.6	73.9	70.0	68.7	69.2	72.8
Acute‐repeated	8.1	9.1	8.7	9.5	11.2	9.9	11.2	12.8	11.4	14.0	11.0
Chronic	0.5	0.5	0.4	0.7	1.5	0.6	0.7	0.7	1.1	1.1	0.8
Acute on chronic	1.1	1.2	1.3	0.8	0.9	0.6	0.9	0.8	1.1	0.9	0.9
Not filled	10.0	11.9	13.9	14.1	14.8	14.3	13.3	15.7	17.7	14.8	14.4
Confirmation criterion											
Clinical‐laboratory	2.3	3.2	3.9	3.3	2.9	3.2	3.2	3.9	3.5	3.3	3.3
Clinical‐epidemiological	19.9	20.5	22.5	24.5	25.7	22.4	23.0	20.5	20.6	20.4	21.9
Clinical	72.6	72.8	70.9	69.0	68.0	71.2	69.9	70.5	71.9	71.5	70.8
Not filled	5.2	3.6	2.7	3.1	3.4	3.2	3.9	5.1	4.0	4.8	4.0
Outcome											
Cure without sequelae	90.2	90.2	88.9	87.0	85.3	84.6	81.3	82.0	83.3	83.1	84.9
Cure with sequelae	0.8	0.5	0.3	1.5	0.8	0.9	1.0	1.1	1.1	0.9	0.9
Death by intoxication	0.4	0.5	0.9	0.5	0.5	0.4	0.6	0.8	0.6	0.4	0.6
Death by other causes	0.1	0.1	0.2	0.2	0.3	0.1	0.3	0.1	0.3	0.2	0.2
Loss to follow‐up	1.6	1.6	1.1	2.0	2.4	1.9	2.5	1.6	2.0	2.1	1.9
Not filled	6.8	7.1	8.5	9.0	10.7	12.1	14.2	14.5	12.7	13.3	11.5

**FIGURE 1:**
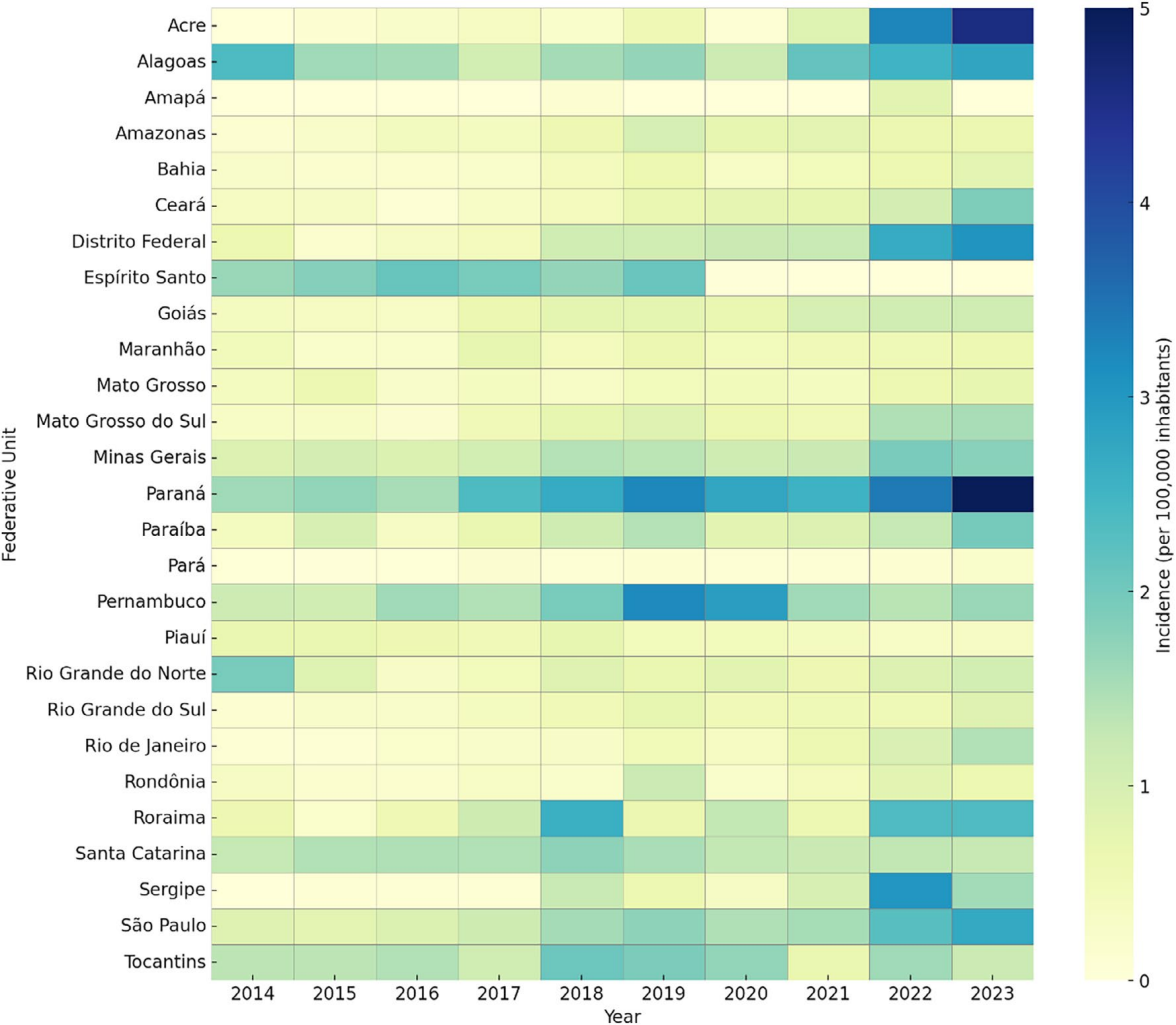
Heatmap showing the incidence rates of self‐medication intoxications in Brazil, by Federative Unit and year (2014–2023). Darker shades indicate higher incidence rates (per 100 000 inhabitants).

Marked disparities were observed in the distribution of notifications across Brazilian FU. São Paulo (28.4%), Paraná (12.8%), and Minas Gerais (11.2%) accounted for the highest volumes of notifications, whereas Rio de Janeiro (3.3%) and Bahia (2.5%) registered lower volumes despite their large population (see Supporting Information for details). National incidence rates of self‐medication‐related intoxications increased from 0.82% in 2014 to 1.87% in 2023. The highest state‐specific incidences in 2023 were observed in Paraná (5.0%), Acre (4.58%), and the Distrito Federal (3.05%) (Figure 1; detailed data available in the Supporting Information).

**FIGURE 2:**
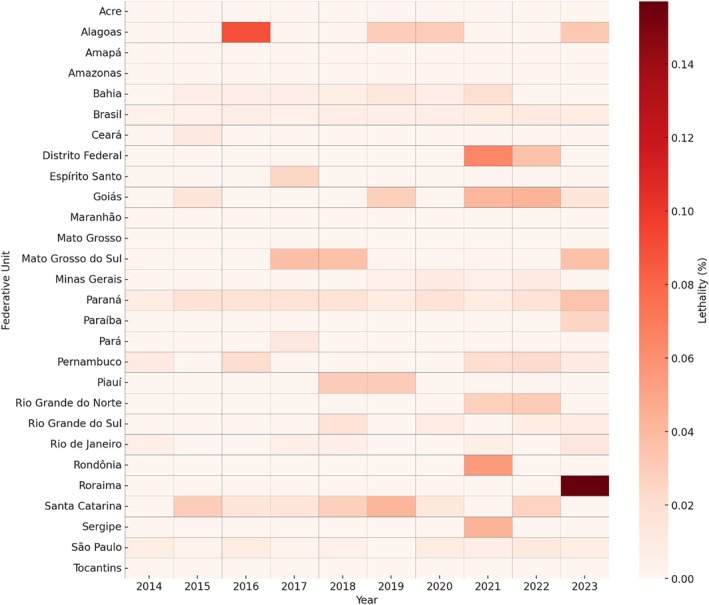
Heatmap showing the lethality rates of self‐medication intoxications in Brazil, by Federative Unit and year (2014–2023). Darker shades indicate higher lethality values (% of deaths among confirmed cases).

The overall lethality rate remained low but rose slightly from 0.003% in 2014 to 0.008% in 2023. A notable outlier was Roraima in 2023, where lethality reached 0.517% (Figure 2; detailed data available in the Supporting Information).

The chi‐squared test revealed statistically significant associations among several pairs of variables, including age and outcome, age and type of exposure, type of exposure and outcome, race and sex, year and type of exposure, sex and outcome, age and race, age and sex, year and sex, and year and race. Notably, all tested variables were associated with at least one other variable, supporting their inclusion in the MCA.

**FIGURE 3:**
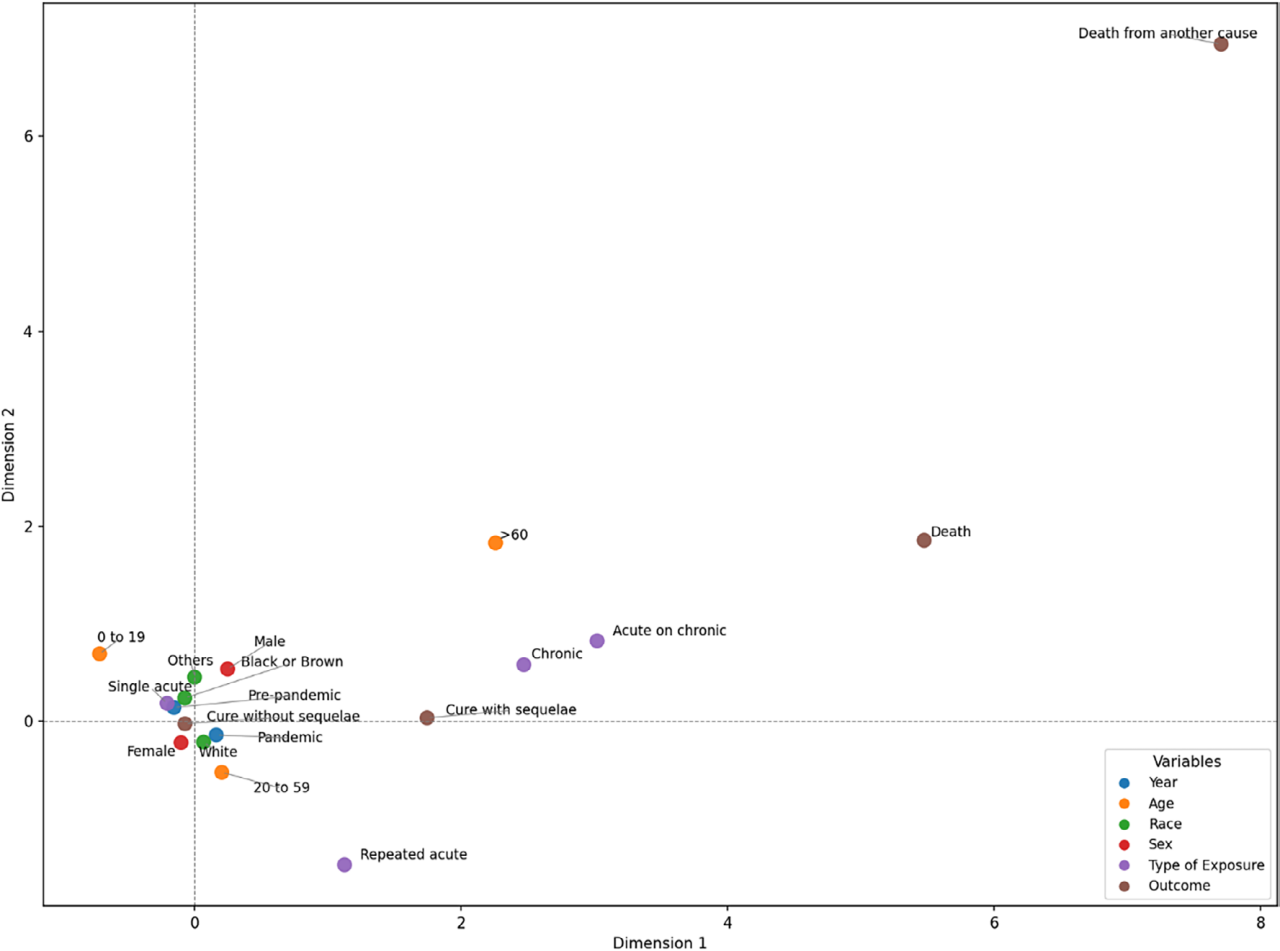
Categories of variables analyzed resulting from the Multiple Correspondence Analysis in self‐medication intoxication cases.

The MCA perceptual map indicated that the first two dimensions collectively accounted for 19.3% of the total inertia (dimension 1 = 10.01%; dimension 2 = 9.28%). Dimension 1 functioned as a severity axis, contrasting less severe cases, characterized by acute single exposures among younger individuals (0–19 years), with more severe cases in older adults (≥ 60 years), involving acute repeated or chronic exposures and were associated with progression to death. Dimension 2 reflected temporal and sociodemographic patterns. A stronger association was observed between the pandemic period and cases involving adult white women (20–59 years), whereas the prepandemic period was more closely linked to black or brown individuals. Acute single exposures were more frequent before the pandemic, whereas repeated and chronic exposures were more frequent during the pandemic. The position of repeated exposures near adults in the perceptual map suggests increased frequency of unregulated medication use in this group (Figure 3).

## 4 Discussion

The nationwide analysis of exogenous intoxications due to self‐medication over a 10‐year period revealed a progressive increase in notifications, particularly from 2022 onward. Several factors likely contributed to this trend, including increased population exposure to self‐medication during the COVID‐19 pandemic, changes in medication use behaviors, and possible improvements in reporting practices [4, 6, 9]. This scenario reflects not only population behavior but also shifts in the culture of adverse event reporting, potentially influenced by heightened awareness of reporting importance during the pandemic.

The predominance of adult individuals, females, and those with intermediate education in the notifications is consistent with previous studies [10, 11] and highlights relevant sociocultural and behavioral factors. Women are often more exposed to self‐medication practices due to their role in family care, broader access to healthcare, and greater exposure to health information and pharmaceutical marketing [12, 13, 14]. The predominance of acute‐single exposure reflects sporadic medication misuse, while the proportional increase in repeated exposures suggests a growing pattern of chronic, unsupervised use that warrants closer attention.

Spatial and temporal analyses revealed marked regional disparities. FU such as São Paulo, Paraná, and Minas Gerais reported the largest number of notifications, while Rio de Janeiro and Bahia, presented disproportionately low notification volumes despite their large populations. These discrepancies may reflect structural inequalities across state health systems, differences in professional training for reporting, and the institutional culture regarding the importance of notification or the epidemiological surveillance [15, 16]. Both quantitative underreporting (nonnotified cases) and qualitative underreporting (incomplete or inconsistent data) are likely contributors.

The increase in self‐medication intoxication incidence, especially during the pandemic period, suggests a direct influence of the sanitary context on medication use behaviors. The widespread dissemination of misinformation and the politicization of unproven treatments, such as ivermectin, hydroxychloroquine, and azithromycin, likely drove indiscriminate drug use [4, 6, 9]. This phenomenon may have been reinforced by growing awareness of the importance of adverse event reporting during the pandemic, particularly linked to vaccine surveillance campaigns.

Although the lethality remained low, the slight increase observed over the study period indicates that severe cases, while minority, pose significant health risks. The association among advanced age, repeated or chronic exposure, and fatal outcomes highlights the vulnerability of older adults, particularly those with multiple comorbidities and prolonged medication use without medical supervision [17].

The MCA provided additional insights, reinforcing two major dimensions: (1) severity, where older adults and repeated or chronic exposures were linked to worse outcomes, and (2) temporal‐sociodemographic variation, with shifts during the pandemic toward adult white women, while prepandemic cases were more associated with black or brown individuals. These findings align with Brazilian studies showing increased irrational self‐medication during the pandemic, especially among women and individuals with higher education or income [10, 11, 18]. The clustering of repeated exposures in adults also underscores concerns about recurrent, unsupervised drug use in this age group. Such practices pose potential health risks, particularly when associated with incorrect information or limited access to healthcare services. These findings reinforce the need for targeted health education, stricter medication regulation, and the strengthening of adverse events reporting culture to prevent intoxications and safeguard public health.

This study has several strengths, including its nationwide scope, large sample size spanning 10 years, and the use of MCA, which allowed for multidimensional exploration of associations between sociodemographic and clinical variables. To our knowledge, this is one of the most comprehensive analyses of self‐medication‐related intoxications in Brazil. However, limitations must be acknowledged. The quality of the data depends on notification practices, which vary across states and are subject to underreporting and incomplete records, particularly regarding education level and type of exposure. Moreover, the use of a secondary source of data limits the quality of information obtained.

The rising incidence of intoxications due to self‐medication in Brazil, coupled with persistent disparities in notification and the increased burden observed during the COVID‐19 pandemic, underscores the urgency of strengthening public health strategies. Efforts should focus on promoting responsible self‐medication, enhancing public health education, and reinforcing surveillance systems to ensure accurate and complete reporting. Policies addressing misinformation and expanding professional training in adverse events reporting are also essential. The promotion of responsible self‐medication, centered on informed autonomy, should be accompanied by strategies to reinforce surveillance and reporting systems, fostering professional training and the intelligent use of data to guide health planning.

### 4.1 Plain Language Summary

Medicines are an essential part of healthcare, but using them without medical guidance—known as self‐medication—can lead to health risks, including intoxication. This study examined all reported cases of intoxication caused by self‐medication in Brazil from 2014 to 2023. We analyzed nearly 24 000 cases reported to the national health system. Most intoxications occurred in women, adults between 20 and 59 years old, and individuals who identified as White or mixed race. In most cases, people recovered fully, but some severe cases, including deaths, were recorded. We found that the number of intoxications increased over the years, with the largest rise occurring during and after the COVID‐19 pandemic. The pandemic also changed who was most affected and how medicines were used, with more cases of repeated or long‐term use without supervision. We also observed strong regional differences: some states reported many cases, while others with large populations reported very few, suggesting gaps in health surveillance. Our findings show that self‐medication is a growing public health concern in Brazil. Improving public education about the safe use of medicines, combating misinformation, and strengthening reporting systems are essential to prevent harmful consequences.

## Ethics Statement

This study did not require approval from a Research Ethics Committee because it was based exclusively on secondary data obtained from the Brazilian Ministry of Health's Notifiable Disease Information System (SINAN).

## Conflicts of Interest

The authors declare no conflicts of interest.

## Supporting information


**Data S1:** pds70269‐sup‐0001‐Supinfo.docx.
