# Supplementary material for: Epidemiological Profile of Exogenous Intoxications by Self‐Medication in Brazil: A Decade of Trends and the Impact of the COVID‐19
Source: Pharmacoepidemiol Drug Saf. 2025 Nov 16;34(11):e70269. doi: 10.1002/pds.70269 (PMC12620163; doi:10.1002/pds.70269)
Supplement: Supplementary file 1 — Data S1: pds70269‐sup‐0001‐Supinfo.docx. [file PDS-34-e70269-s001.docx]

**Supplementary Material**

Frequency of self-medication poisonings between 2014 and 2023 in Brazil by Federative Unit

| Federative Unity | Frequency (%) | | | | | | | | | | |  |
| --- | --- | --- | --- | --- | --- | --- | --- | --- | --- | --- | --- | --- |
|  |  |  |  |  |  |  |  |  |  |  |  |  |
|  | 2014 | 2015 | 2016 | 2017 | 2018 | 2019 | 2020 | 2021 | 2022 | 2023 | 2014-2023 |  |
| Acre | 0.0 | 0.1 | 0.1 | 0.2 | 0.1 | 0.2 | 0.0 | 0.3 | 0.9 | 1.0 | 0.4 |  |
| Alagoas | 4.8 | 3.5 | 3.3 | 1.8 | 2.1 | 1.9 | 1.7 | 3.0 | 2.5 | 2.3 | 2.5 |  |
| Amapá | 0.0 | 0.0 | 0.0 | 0.0 | 0.0 | 0.0 | 0.0 | 0.0 | 0.2 | 0.0 | 0.0 |  |
| Amazonas | 0.3 | 0.7 | 1.1 | 0.8 | 1.0 | 1.4 | 1.3 | 1.4 | 0.8 | 0.7 | 1.0 |  |
| Bahia | 2.3 | 1.7 | 1.7 | 1.8 | 2.6 | 3.2 | 2.1 | 2.8 | 2.8 | 3.0 | 2.5 |  |
| Ceará | 1.9 | 2.0 | 0.6 | 1.4 | 1.6 | 2.2 | 3.0 | 2.9 | 2.9 | 4.3 | 2.5 |  |
| Distrito Federal | 1.1 | 0.3 | 0.6 | 0.7 | 1.3 | 1.1 | 1.6 | 1.6 | 2.4 | 2.3 | 1.5 |  |
| Espírito Santo | 3.9 | 4.7 | 5.3 | 4.0 | 2.7 | 2.8 | 0.0 | 0.0 | 0.0 | 0.0 | 1.9 |  |
| Goiás | 1.5 | 1.6 | 1.4 | 2.3 | 2.2 | 1.9 | 2.1 | 3.1 | 2.4 | 2.0 | 2.1 |  |
| Maranhão | 1.9 | 1.1 | 0.9 | 2.6 | 1.2 | 1.6 | 1.4 | 1.6 | 1.2 | 1.1 | 1.4 |  |
| Mato Grosso | 0.8 | 1.3 | 0.6 | 0.7 | 0.4 | 0.5 | 0.7 | 0.6 | 0.7 | 0.7 | 0.7 |  |
| Mato Grosso do Sul | 0.5 | 0.5 | 0.3 | 0.7 | 0.8 | 0.8 | 0.8 | 0.6 | 1.3 | 1.1 | 0.8 |  |
| Minas Gerais | 11.4 | 14.4 | 12.4 | 11.5 | 11.9 | 9.6 | 10.2 | 11.0 | 12.4 | 9.6 | 11.2 |  |
| Pará | 0.2 | 0.1 | 0.3 | 0.6 | 0.3 | 0.3 | 0.4 | 0.3 | 0.3 | 0.5 | 0.3 |  |
| Paraíba | 1.0 | 2.6 | 0.9 | 1.4 | 1.8 | 1.9 | 1.4 | 1.6 | 1.6 | 2.0 | 1.7 |  |
| Paraná | 10.7 | 12.6 | 10.8 | 13.7 | 12.1 | 12.4 | 13.7 | 12.5 | 12.3 | 15.0 | 12.8 |  |
| Pernambuco | 6.6 | 6.7 | 9.5 | 6.9 | 7.4 | 10.3 | 12.0 | 6.5 | 3.9 | 3.9 | 7.1 |  |
| Piauí | 1.3 | 1.5 | 1.2 | 0.9 | 1.0 | 0.5 | 0.6 | 0.6 | 0.3 | 0.3 | 0.7 |  |
| Rio de Janeiro | 0.8 | 0.9 | 2.2 | 2.1 | 2.0 | 2.9 | 2.7 | 4.6 | 4.9 | 6.1 | 3.3 |  |
| Rio Grande do Norte | 4.0 | 2.0 | 0.6 | 0.8 | 1.2 | 0.8 | 1.2 | 0.9 | 0.9 | 0.9 | 1.2 |  |
| Rio Grande do Sul | 0.8 | 1.9 | 2.0 | 2.3 | 2.4 | 2.8 | 2.5 | 2.7 | 1.9 | 2.5 | 2.3 |  |
| Rondônia | 0.4 | 0.2 | 0.2 | 0.3 | 0.2 | 0.7 | 0.2 | 0.3 | 0.4 | 0.3 | 0.3 |  |
| Roraima | 0.2 | 0.1 | 0.2 | 0.3 | 0.6 | 0.1 | 0.3 | 0.2 | 0.5 | 0.4 | 0.3 |  |
| Santa Catarina | 5.1 | 6.4 | 6.3 | 5.1 | 4.9 | 3.6 | 4.0 | 3.8 | 3.1 | 2.5 | 4.1 |  |
| São Paulo | 23.1 | 23.4 | 26.3 | 26.1 | 28.2 | 26.8 | 28.9 | 30.4 | 31.4 | 31.8 | 28.4 |  |
| Sergipe | 0.0 | 0.1 | 0.1 | 0.1 | 1.1 | 0.5 | 0.3 | 1.0 | 2.1 | 0.9 | 0.8 |  |
| Tocantins | 1.2 | 1.3 | 1.4 | 0.9 | 1.3 | 1.0 | 1.2 | 0.5 | 0.8 | 0.5 | 0.9 |  |
| Not filled | 14.4 | 8.3 | 9.7 | 10.1 | 7.8 | 8.1 | 5.7 | 5.3 | 5.1 | 4.3 | 7.3 |  |
| Total | 100.0 | 100.0 | 100.0 | 100.0 | 100.0 | 100.0 | 100.0 | 100.0 | 100.0 | 100.0 | 100.0 |  |

Source: Original research results

Incidence of self-medication poisonings between 2014 and 2023 in Brazil by Federative Unit

| Federative Unit | Incidence of self-medication poisonings (%) | | | | | | | | | |
| --- | --- | --- | --- | --- | --- | --- | --- | --- | --- | --- |
|  | 2014 | 2015 | 2016 | 2017 | 2018 | 2019 | 2020 | 2021 | 2022 | 2023 |
| Acre | 0.00 | 0.13 | 0.24 | 0.36 | 0.23 | 0.57 | 0.11 | 0.88 | 3.25 | 4.58 |
| Alagoas | 2.38 | 1.60 | 1.55 | 1.07 | 1.56 | 1.68 | 1.16 | 2.14 | 2.53 | 2.78 |
| Amapá | 0.00 | 0.00 | 0.00 | 0.00 | 0.12 | 0.00 | 0.00 | 0.00 | 0.82 | 0.00 |
| Amazonas | 0.13 | 0.26 | 0.42 | 0.39 | 0.59 | 1.01 | 0.71 | 0.80 | 0.66 | 0.66 |
| Bahia | 0.25 | 0.17 | 0.18 | 0.23 | 0.43 | 0.64 | 0.32 | 0.45 | 0.64 | 0.80 |
| Ceará | 0.36 | 0.34 | 0.11 | 0.31 | 0.44 | 0.70 | 0.75 | 0.74 | 1.05 | 1.88 |
| Distrito Federal | 0.63 | 0.18 | 0.34 | 0.43 | 1.11 | 1.09 | 1.21 | 1.23 | 2.66 | 3.05 |
| Espírito Santo | 1.65 | 1.83 | 2.11 | 1.94 | 1.69 | 2.09 | 0.02 | 0.00 | 0.00 | 0.00 |
| Goiás | 0.38 | 0.37 | 0.33 | 0.66 | 0.78 | 0.78 | 0.67 | 1.01 | 1.08 | 1.11 |
| Maranhão | 0.47 | 0.23 | 0.22 | 0.73 | 0.43 | 0.66 | 0.46 | 0.52 | 0.55 | 0.62 |
| Mato Grosso | 0.40 | 0.62 | 0.27 | 0.39 | 0.32 | 0.46 | 0.48 | 0.39 | 0.63 | 0.71 |
| Mato Grosso do Sul | 0.31 | 0.31 | 0.15 | 0.52 | 0.73 | 0.86 | 0.64 | 0.53 | 1.45 | 1.52 |
| Minas Gerais | 0.91 | 1.05 | 0.93 | 1.07 | 1.41 | 1.35 | 1.12 | 1.21 | 1.92 | 1.78 |
| Pará | 0.04 | 0.02 | 0.05 | 0.14 | 0.08 | 0.12 | 0.10 | 0.08 | 0.12 | 0.23 |
| Paraíba | 0.41 | 0.99 | 0.35 | 0.67 | 1.13 | 1.42 | 0.82 | 0.91 | 1.26 | 1.96 |
| Paraná | 1.60 | 1.71 | 1.52 | 2.38 | 2.68 | 3.24 | 2.77 | 2.55 | 3.41 | 5.00 |
| Pernambuco | 1.17 | 1.09 | 1.59 | 1.44 | 1.94 | 3.21 | 2.91 | 1.59 | 1.38 | 1.66 |
| Piauí | 0.69 | 0.69 | 0.59 | 0.53 | 0.74 | 0.46 | 0.46 | 0.40 | 0.31 | 0.34 |
| Rio de Janeiro | 0.08 | 0.08 | 0.20 | 0.25 | 0.29 | 0.49 | 0.36 | 0.62 | 0.97 | 1.44 |
| Rio Grande do Norte | 1.94 | 0.88 | 0.29 | 0.46 | 0.86 | 0.68 | 0.82 | 0.59 | 0.91 | 1.06 |
| Rio Grande do Sul | 0.12 | 0.25 | 0.27 | 0.41 | 0.54 | 0.73 | 0.52 | 0.55 | 0.56 | 0.87 |
| Rondônia | 0.34 | 0.17 | 0.17 | 0.33 | 0.23 | 1.18 | 0.22 | 0.44 | 0.82 | 0.63 |
| Roraima | 0.60 | 0.20 | 0.58 | 1.15 | 2.60 | 0.66 | 1.27 | 0.61 | 2.36 | 2.36 |
| Santa Catarina | 1.25 | 1.44 | 1.45 | 1.43 | 1.74 | 1.49 | 1.28 | 1.21 | 1.29 | 1.24 |
| São Paulo | 0.87 | 0.80 | 0.93 | 1.14 | 1.55 | 1.74 | 1.46 | 1.54 | 2.25 | 2.72 |
| Sergipe | 0.00 | 0.09 | 0.09 | 0.09 | 1.23 | 0.61 | 0.35 | 0.98 | 3.03 | 1.58 |
| Tocantins | 1.34 | 1.34 | 1.44 | 1.10 | 2.06 | 1.91 | 1.70 | 0.68 | 1.59 | 1.19 |
| Brazil | 0.82 | 0.74 | 0.77 | 0.95 | 1.20 | 1.41 | 1.10 | 1.11 | 1.56 | 1.87 |

Source: Original research results

Lethality rate of self-medication poisonings between 2014 and 2023 in Brazil, by Federative Unit

| Federative Unit | Lethality per year (%) | | | | | | | | | |
| --- | --- | --- | --- | --- | --- | --- | --- | --- | --- | --- |
|  |  |  |  |  |  |  |  |  |  |  |
|  | 2014 | 2015 | 2016 | 2017 | 2018 | 2019 | 2020 | 2021 | 2022 | 2023 |
| Acre | 0.000 | 0.000 | 0.000 | 0.000 | 0.000 | 0.000 | 0.000 | 0.000 | 0.000 | 0.000 |
| Alagoas | 0.000 | 0.000 | 0.089 | 0.000 | 0.000 | 0.030 | 0.030 | 0.000 | 0.000 | 0.032 |
| Amapá | 0.000 | 0.000 | 0.000 | 0.000 | 0.000 | 0.000 | 0.000 | 0.000 | 0.000 | 0.000 |
| Amazonas | 0.000 | 0.000 | 0.000 | 0.000 | 0.000 | 0.000 | 0.000 | 0.000 | 0.000 | 0.000 |
| Bahia | 0.000 | 0.007 | 0.007 | 0.007 | 0.007 | 0.013 | 0.007 | 0.020 | 0.000 | 0.000 |
| Ceará | 0.000 | 0.011 | 0.000 | 0.000 | 0.000 | 0.000 | 0.000 | 0.000 | 0.000 | 0.000 |
| Distrito Federal | 0.000 | 0.000 | 0.000 | 0.000 | 0.000 | 0.000 | 0.000 | 0.065 | 0.035 | 0.000 |
| Espírito Santo | 0.000 | 0.000 | 0.000 | 0.025 | 0.000 | 0.000 | 0.000 | 0.000 | 0.000 | 0.000 |
| Goiás | 0.000 | 0.015 | 0.000 | 0.000 | 0.000 | 0.028 | 0.000 | 0.042 | 0.043 | 0.014 |
| Maranhão | 0.000 | 0.000 | 0.000 | 0.000 | 0.000 | 0.000 | 0.000 | 0.000 | 0.000 | 0.000 |
| Mato Grosso | 0.000 | 0.000 | 0.000 | 0.000 | 0.000 | 0.000 | 0.000 | 0.000 | 0.000 | 0.000 |
| Mato Grosso do Sul | 0.000 | 0.000 | 0.000 | 0.037 | 0.036 | 0.000 | 0.000 | 0.000 | 0.000 | 0.036 |
| Minas Gerais | 0.000 | 0.000 | 0.000 | 0.000 | 0.000 | 0.005 | 0.009 | 0.005 | 0.010 | 0.000 |
| Pará | 0.000 | 0.000 | 0.000 | 0.012 | 0.000 | 0.000 | 0.000 | 0.000 | 0.000 | 0.000 |
| Paraíba | 0.000 | 0.000 | 0.000 | 0.000 | 0.000 | 0.000 | 0.000 | 0.000 | 0.000 | 0.025 |
| Paraná | 0.009 | 0.018 | 0.018 | 0.018 | 0.018 | 0.009 | 0.017 | 0.009 | 0.017 | 0.035 |
| Pernambuco | 0.011 | 0.000 | 0.021 | 0.000 | 0.000 | 0.000 | 0.000 | 0.021 | 0.022 | 0.011 |
| Piauí | 0.000 | 0.000 | 0.000 | 0.000 | 0.031 | 0.031 | 0.000 | 0.000 | 0.000 | 0.000 |
| Rio de Janeiro | 0.006 | 0.000 | 0.000 | 0.006 | 0.006 | 0.000 | 0.000 | 0.006 | 0.000 | 0.012 |
| Rio Grande do Norte | 0.000 | 0.000 | 0.000 | 0.000 | 0.000 | 0.000 | 0.000 | 0.028 | 0.030 | 0.000 |
| Rio Grande do Sul | 0.000 | 0.000 | 0.000 | 0.000 | 0.018 | 0.000 | 0.009 | 0.000 | 0.009 | 0.009 |
| Rondônia | 0.000 | 0.000 | 0.000 | 0.000 | 0.000 | 0.000 | 0.000 | 0.055 | 0.000 | 0.000 |
| Roraima | 0.000 | 0.000 | 0.000 | 0.000 | 0.000 | 0.000 | 0.000 | 0.000 | 0.000 | 0.157 |
| Santa Catarina | 0.000 | 0.030 | 0.014 | 0.014 | 0.028 | 0.042 | 0.014 | 0.000 | 0.026 | 0.000 |
| São Paulo | 0.007 | 0.002 | 0.009 | 0.002 | 0.004 | 0.000 | 0.009 | 0.006 | 0.011 | 0.007 |
| Sergipe | 0.000 | 0.000 | 0.000 | 0.000 | 0.000 | 0.000 | 0.000 | 0.043 | 0.000 | 0.000 |
| Tocantins | 0.000 | 0.000 | 0.000 | 0.000 | 0.000 | 0.000 | 0.000 | 0.000 | 0.000 | 0.000 |
| Not filled |  |  |  |  |  |  |  |  |  |  |
| Brazil | 0.003 | 0.004 | 0.007 | 0.004 | 0.006 | 0.006 | 0.006 | 0.009 | 0.010 | 0.008 |

Source: Original research results
